# *Levilactobacillus brevis* 47f: Bioadaptation to Low Doses of Xenobiotics in Aquaculture

**DOI:** 10.3390/biology13110925

**Published:** 2024-11-14

**Authors:** Diana Reznikova, Nikita Kochetkov, Alexey Vatlin, Dmitry Nikiforov-Nikishin, Olesya Galanova, Anastasia Klimuk, Svetlana Smorodinskaya, Daria Matyushkina, Alexey Kovalenko, Ivan Butenko, Maria Marsova, Valery Danilenko

**Affiliations:** 1 Laboratory of Bacterial Genetics, Vavilov Institute of General Genetics, Russian Academy of Sciences, 119333 Moscow, Russia; 9150699@mail.ru (N.K.); vatlin_alexey123@mail.ru (A.V.); niknikdl@rambler.ru (D.N.-N.); olesyagalanova2001@yandex.ru (O.G.); klimukanastasia27@gmail.com (A.K.); kler.smo@gmail.com (S.S.); masha_marsova@mail.ru (M.M.); valerid@vigg.ru (V.D.); 2 Moscow Center for Advanced Studies, 20, Kulakova Str., 123592 Moscow, Russia; 3 Faculty of Biotechnology and Fisheries, Moscow State University of Technologies and Management (FCU), 73, Zemlyanoy Val Str., 109004 Moscow, Russia; 4 Institute of Ecology, Peoples’ Friendship University of Russia (RUDN University), 117198 Moscow, Russia; 5 Scientific Research Institute for Systems Biology and Medicine, Scientific Driveway, 18, 117246 Moscow, Russia

**Keywords:** *Levilactobacillus brevis* 47f, bioadaptation, xenobiotics, *Danio rerio*, cytokine profile, transcriptome, proteome

## Abstract

Agricultural and industrial activities are increasing pollution of water bodies with low doses of xenobiotics that have detrimental effects on aquaculture. The aim of this work was to determine the possibility of using *Levilactobacillus brevis* 47f culture in fish aquaculture under the influence of low doses of xenobiotics as an adaptogen. An increase in the survival of *Danio rerio* individuals exposed to the xenobiotic bisphenol A solution and fed with the *L. brevis* 47f was shown compared to control groups and, at the same time, the cytokine profile in the intestinal tissues of *Danio rerio* was also investigated. Analysis of differential gene expression of the *L. brevis* 47f grown under the action of high concentrations of bisphenol A showed changes in mRNA levels of a number of genes, including genes of various transport proteins, genes involved in fatty acid synthesis, genes of transcriptional regulators, genes of the arabinose operon, and the *oppA* gene. The identification of *L. brevis* 47f proteins from polyacrylamide gel by mass spectrometry revealed L-arabinose isomerase, Clp chaperone subunit, ATP synthase subunits, pentose phosphate pathway and glycolysis enzyme proteins, which are likely part of the *L. brevis* 47f strain’s anti-stress response, but probably do not affect its adaptogenic activity toward *Danio rerio*.

## Simple Summary

Environmental pollution by low doses of xenobiotic is an important problem, including for aquaculture. To reduce the effects of constant stress, probiotics are often used as functional feed additives in aquaculture. This study investigates the possibility of using the strain *Levilactobacillus brevis* 47f as an adaptogen in aquaculture using the model organism zebrafish (*Danio rerio*). The cytokine profile in intestinal tissues of *Danio rerio* against the background of their exposure to xenobiotic solution and the fact of receiving the *L. brevis* 47f was examined. The transcriptome profile of the *L. brevis* 47f was also studied and the proteins that change their quantity against stress were revealed, which makes it possible to suggest that bisphenol A does not affect the adaptogenic properties of the *L. brevis* 47f.

## 1. Introduction

Nowadays, our environment is strongly influenced by industrial and agricultural impact. In this connection, environmental pollution is increasing, the content of xenobiotics—chemical substances foreign to living organisms—in soil, water bodies and, as a consequence, in human foodstuffs is also growing [1]. In particular, aquaculture is susceptible to the influence of xenobiotics. Chronic exposure of fish individuals to low concentrations of xenobiotics leads to the development of diseases associated with various body systems [2,3,4]. To reduce the negative effects of xenobiotics and prevent diseases in aquaculture, antibiotics are often used, but their use in uncontrolled amounts can lead to resistance [5]. Therefore, the field of more environmentally friendly feed additives that can partially replace antibiotics with similar protective functions is being actively developed.

Bisphenol A was used as a xenobiotic in this study. Bisphenol A is actively used in the production of technological plastic products [6]. Bisphenol A, entering water bodies through the effluents of sewage treatment plants and factories, has a negative impact when present at physiologically relevant concentrations on the endocrine [2], nervous, digestive, and excretory systems [4] of hydrobionts [7]. In addition, bisphenol A has a significant impact on the reproductive system due to its estrogenic activity [3].

The microbiome is a source of biologically active compounds, which may include primary (e.g., short-chain fatty acids derived from the processing of carbohydrates), secondary metabolites, molecular structures associated with microorganisms (their components), and antigens derived from the microbiome. For example, short-chain fatty acids regulate the differentiation of immune cells in the gut itself, while microbiome-derived antigens can cross-react with anti-pathogenic, autoimmune or tumor-associated antigens throughout the body [8]. Secondary metabolites are low molecular weight compounds, products of bacterial metabolism of antibiotics, pigments, toxins, enzyme inhibitors, immunomodulators, and neuromodulators, which have their own biological activity and can affect the host organism [9]. Due to the secretion of various bioactive substances by microorganisms, the idea of using them as pharmabiotics and postbiotics arises [10,11]. The new trend in the field of probiotics includes the development of live biotherapeutics and pharmabiotics for which the molecular mechanisms of their action have been elucidated [10,12,13]. Thus, a probiotic strain with proven properties and mechanisms can act as a possible drug to increase the immune response of fish, as well as their ability to adapt to external influences and avoid the deleterious effects of xenobiotics.

In fish, as in humans and other mammals, the intestinal microbiota is very diverse and is represented by a huge number of microorganisms [14]. In humans, the predominant types are *Firmicutes* and *Bacteroidetes*; in contrast, in fish, *Proteobacteria* and *Fusobacteria* are the most common [15]. Studies of microbiota–host interactions have shown that commensal bacteria can influence and regulate various physiological processes of the organism. It has been shown that the microbiota controls feeding behavior, energy balance, nutrient metabolism, immune response and more [16]. These studies are also of practical nature applicable to aquaculture, which has faced problems such as deterioration of water quality, excessive use of antibiotics [17], various diseases related to nervous system, cardiovascular system, abnormal development of organs of aquaculture subjects [18]. Understanding and knowing which body systems are affected by the microbiota are very important tasks because by changing the composition of the microbiota, the entire fish body can be influenced. The use of probiotics in aquaculture helps to improve farmed fish growth, immunity, integrity of the intestinal epithelial barrier, reduction of side effects of antibiotics and consequently its ability to resist various diseases [19,20]. In addition, probiotics can be a good alternative to the use of antibiotics in aquaculture [21].

The *Levilactobacillus brevis* 47f strain was isolated from the organism of a healthy resident of the Central European part of the Russian Federation. In many studies, the *L. brevis* 47f has been shown to possess the antioxidant [22], adaptogenic [23], anti-inflammatory [24] properties in rats and mice. Since the *Levilactobacillus brevis* 47f strain has been isolated from the human body, its use as an adaptogen for aquaculture should not pose a threat to human health [25].

Omics technologies allow obtaining comprehensive information about the functioning at the molecular and genetic level of the object under study due to the ability to identify the molecules involved in these processes. The omics approach to the study of biological samples is a very broad concept. It includes genomics (detection of genes), transcriptomics (RNA analysis), proteomics, and metabolomics (determination of the spectrum of proteins and metabolites, respectively). The main objective of using omics technologies in the modern world is to study a biological system as a whole to fully understand the processes occurring in it, tracing their complete pathway from genes to proteins and metabolites [26]. Since the intestinal microbiota is a complex system, the application of an integrated approach using omics technologies becomes just right for its study. Omics technologies are also used to identify molecular mechanisms of probiotic action [27].

The aim of this work was to establish the possibility of using *Levilactobacillus brevis* 47f culture in fish aquaculture when exposed to low doses of xenobiotics as an adaptogen and determination of xenobiotic effect on strain viability using omics technologies. Using qPCR methods, changes in cytokine profile in intestinal tissues of *Danio rerio* exposed to bisphenol A and receiving and not receiving *L. brevis* 47f strain were analyzed. Using transcriptomic and proteomic analysis of the *L. brevis* 47f grown in the presence of different doses of xenobiotic, the effect of the toxicant on strain viability was evaluated.

## 2. Materials and Methods

### 2.1. Bacterial Strain

The strain *Levilactobacillus brevis* 47f (NCBI BioProject PRJNA280953), belonging to the collection of the Laboratory of Microbial Genetics of the Vavilov Institute of General Genetics of the Russian Academy of Sciences, was isolated from the body of a healthy woman living in Central Russia. The strain was deposited in the All-Russian Collection of Industrial Microorganisms under the number B-12237.

### 2.2. Conditions of Bacterial Strain Cultivation, Lyophilization

*Levilactobacillus brevis* 47f strain was grown in both liquid and agar MRS medium (HiMedia, Mumbai, India) at 37 °C in a desiccator, in which oxygen was burned by candle flame. MRS liquid medium contains proteose peptone 10 g/L, beaf extract 10 g/L, yeast extract 5 g/L, dextrose 20 g/L, tween-80 1 g/L, ammonium citrate 2 g/L, sodium acetate 5 g/L, magnesium sulfate 0.1 g/L, manganese sulfate 0.05 g/L, sodium hydrophosphate 2 g/L. In MRS solid agar medium, the composition is identical to the liquid medium except for the replacement of sodium hydrophosphate with potassium hydrophosphate 2 g/L, and the addition of agar-agar 12 g/L. 

For lyophilization, the 18 h culture (10^9^ CFU/mL) was centrifuged at 7000× *g* at 4 °C for 10 min, and then washed with PBS buffer (potassium dihydrophosphate 1.7 mM, sodium dihydrophosphate 5.2 mM, sodium chloride 150 mM, pH = 7.4) and resuspended in lyophilization medium (10% sucrose, 1% gelatin). The mixture was kept at −20 °C for 24 h and then dried at −52 °C and 0.42 mbar pressure for 48 h on a Labconco 2.5 lyophilic dryer (Labconco, Kansas City, MO, USA). The obtained lyophilisates were stored at 4 °C. Their viability and titer were checked before use in the experiment. 

### 2.3. Animal Collection and Maintenance 

All animal experiments were conducted at the Department of Biotechnology and Fisheries of the Moscow State University of Technology and Management in accordance with the European Directive 2010/63/EU of the European Parliament as translated into Russian (Council of 22 September 2010 on the protection of animals used for scientific purposes) and were approved by the Local Ethics Commission of the Scientific and Technical Council of the Moscow State University of Technology and Management (approval number 1, 15 January 2023).

All experiment designs were made in clear accordance with the 3R principles:

Replacement: the use of lower organisms was not possible due to the need to study complex biochemical, morphological and behavioral changes, as well as the specificity of the action of the substances used specifically on the fish organism. 

Reduction: the number of animals in the group was strictly regulated by this biotic principle and did not exceed 20 animals, which was sufficient to obtain statistically significant differences (if any) in the planned tests.

Refinement: animals were kept in specially equipped rooms, fully equipped with the necessary equipment and qualified personnel, with appropriate routine evaluation of the condition.

Wild-type *Danio rerio* individuals 4 months old, with average size of 2.12 ± 0.3 cm and weight of 0.29 ± 0.03 g, were used in the experiment. They were kept in aquariums of 300 L with mechanical and biological filtration systems and daily 10% water change. Prior to the experiment, individuals were fed with Tetra Min Flakes XL («Tetra», Melle, Germany). Healthy individuals without visible changes were selected for the study. Twenty individuals of both sexes were kept in 50 L tanks with constant aeration. Temperature (24 °C), light regime (12 h darkness/12 h light), and hydrochemical parameters (pH 7.2 ± 0.2; O_2_ 7.8 ± 0.3 mg/L; NH_4_ < 0.05 mg/L; NO_2_ 0.2 ± 0.01 mg/L; NO_3_ 5.3 ± 1.1 mg/L) were kept identical to those in the tanks before the experiment. Feeding occurred ad libitum. 

### 2.4. Experimental Design

Bisphenol A (2,2-bis(4-hydroxyphenyl)propane) (Sigma-Aldrich, St. Louis, MO, USA) was considered as a model toxicant in the experiment. The concentration of bisphenol A was chosen to be 2 mg/L because this is the concentration that can potentially be detected in real water bodies [28,29]. Toxicant solution was prepared immediately before placing them in the aquariums. The water in the experimental groups was changed every two days to ensure the stability of the toxicant concentration. 

Four groups were involved in the experiment. The control group (CTR) received basic feed without any additives and was in a toxicant-free solution, the BPA group received basic feed and was in a bisphenol A solution, the LAC group received a *Levilactobacillus brevis* 47f additive with feed and was in a toxicant-free solution, the BPL group received the *L. brevis* 47f under consideration with feed and was in a bisphenol A solution. The duration of the chronic experiment was 60 days. The survival rate of individuals was monitored daily throughout the experiment. 

### 2.5. Feed Preparation

Coppens Scarlet 0.5–0.8 mm [protein 53%, fat 13%, fiber 0.7%, ash 8.8%] (Coppens, Helmond, The Netherlands) was used as the main feed in the experiment. The *L. brevis* 47f strain was added to the feed by encapsulation using sodium alginate (Ruskhim, Moscow, Russia). Sodium alginate was dissolved in distilled water (0.5 g per 100 mL of water), and then a lyophilized culture of *Levilactobacillus brevis* 47f at a concentration of 1 × 10^8^ CFU/g was added to the resulting solution. The mixture was thoroughly mixed and applied to the feed using a high-pressure pump spray («Santrade Plastics Group Inc.», Markham, ON, Canada). Then, it was dried at 4 °C for 15 h, and further storage of the feed was performed at 4 °C. Before adding the *L. brevis* 47f to the feed, it was tested for viability by sowing on MRS solid agar medium (HiMedia, Mumbai, India) and counting CFU. Sodium alginate was also added to the feed of individuals not receiving the *L. brevis* 47f strain in the same quantities as the probiotic supplemented feed. To maintain the probiotic properties and quality of the feed, it was reconstituted every two weeks. 

### 2.6. RNA Isolation from Danio rerio Intestinal Tissue

After the end of the experiment on day 60, three individuals without external lesions were taken from each experimental group. After anesthesia with MS-222 solution (10 mg/L), intestinal tissues were taken for subsequent RNA extraction. RNA was then isolated using ExtractRNA reagent («Eurogen», Moscow, Russia) according to the manufacturer’s instructions. The concentrations of isolated RNA were measured using a Qubit fluorimeter (Invitrogen, Waltham, MA, USA).

### 2.7. RNA Preparation and Reverse Transcription Reaction 

DNAase E («Eurogen», Moscow, Russia) was used to remove the remaining genomic DNA from the samples according to the manufacturer’s instructions. RNA quality was checked by agarose gel electrophoresis. The reverse transcription reaction was performed using the MMLV RT kit («Eurogen», Moscow, Russia) according to the manufacturer’s instructions.

### 2.8. Real-Time PCR

For real-time PCR, 1 ng of total cDNA was taken. The reaction mixture was prepared using qPCRmix-HS SYBR kit («Eurogen», Moscow, Russia) according to the manufacturer’s recommendations. RT-PCR was performed on a CFX96 (Bio-Rad, Hercules, CA, USA). The amplification program was carried out as follows: melting—5 min, 95 °C, denaturation—30 s, 95 °C, annealing—30 s, 60 °C, elongation—30 s, 72 °C. The last three steps were repeated cyclically 50 times, and then the DNA melting curve was obtained. CFX Manager V 3.1 software (Bio-Rad, Hercules, CA, USA) was used for RT-PCR data analysis, relative normalized expressions in three biological replicates (three independent individuals per point) were calculated as ∆∆Cq, and the housekeeping gene *actb1* was taken as a reference [30]. Primers were selected using primer-BLAST [31] (Table A1). 

### 2.9. Sample Preparation for Transcriptome Analysis of Levilactobacillus brevis 47f

For transcriptome analysis, the bacterial strain *Levilactobacillus brevis* 47f was crossed from MRS solid agar medium (HiMedia, Mumbai, India) to liquid MRS medium (HiMedia, Mumbai, India). After 18 h, the culture was transferred at a ratio of 1:50 and addition of bisphenol A to the culture was performed so that the final concentrations of toxicant in the solutions were 0, 2, 50 mg/L. The final volume was 10 mL. Cultures were prepared in three biological replicates and grown to mid-exponential phase (OD600 = 0.8) at 37 °C. 

The bacterial cells were then separated from the culture fluid by centrifugation at 7000× *g* for 5 min at 4 °C. Next, RNAprotect (QIAGEN, Gardena, CA, USA) was added to the precipitate and washed by centrifugation at 7000× *g* for 5 min at 4 °C. The supernatant was removed, ExtractRNA ((«Eurogen», Moscow, Russia) was added to the precipitate, the contents were transferred to Lising matrix B tubes (ThermoFisher Scientific, Waltham, MA, USA), and then homogenization was performed using a SpeedMill plus device («analyticjena», Jena, Germany). RNA was then isolated using ExtractRNA reagent ((«Eurogen», Moscow, Russia) according to the manufacturer’s instructions.

Total RNA (250 ng) was used for library preparation. Ribosomal RNA was removed from the total RNA using the Ribo-Zero Plus rRNA Depletion Kit (Illumina, San Diego, CA, USA) and libraries were prepared using the KAPA RNA Hyper Kit (Roche, Switzerland), according to the manufacturer protocol. Subsequently, RNA cleanup was performed with the RNA Clean XP kit (Beckman Coulter, Brea, CA, USA). The library underwent a final cleanup using the KAPA HyperPure Beads (Roche, CH-4070 Basel, Switzerland) after which the libraries’ size distribution and quality were assessed using a high sensitivity DNA chip (Agilent Technologies, Santa Clara, CA, USA). Libraries were subsequently quantified by Quant-iT DNA Assay Kit, High Sensitivity (Thermo Fisher Scientific, Waltham, MA, USA). Finally, equimolar quantities of all libraries (10 pM) were sequenced by a high throughput run on the Illumina HiSeq 2500 using 2 × 100 bp reads and a 2% Phix spike-in control.

### 2.10. Analysis of RNAseq Data 

Quality of FASTQ files was assessed using FastQC v0.12.1. Reads were mapped to the reference *Levilactobacillus brevis* 47f genome (GCF_001010995.1) using STAR v2.7.11a [32]. Mapping quality control metrics (QC) were obtained with samtools v1.17 [33], mosdepth v0.3.4 [34], and QualiMap v.2.2.2-dev [35], and subsequently MultiQC v1.17 [36] was used to aggregate all QC metrics. Gene counts were obtained using the featureCounts v2.0.6 [37]. Differential gene expression analysis was performed with the edgeR v3.42.4 [38] package using quasi-likelihood F-test. The data were uploaded to NCBI (BioProject PRJNA1121097).

### 2.11. Sample Preparation for Proteomic Analysis of Levilactobacillus brevis 47f

Culture growth of *Levilactobacillus brevis* 47f for subsequent proteomic analysis was carried out in a similar manner to sample preparation for transcriptome analysis, except only the culture in the presence of toxin was grown for 21 h to stationary growth phase (OD600 = 1.7) to allow time for the production of all necessary proteins. Bacterial cells were separated from the culture fluid by centrifugation at 7000× *g* for 10 min at 4 °C. The cells were then washed 3 times with a mixture of PBS and PMSF (1:100), centrifuging at 7000× *g* for 10 min at 4 °C each time after addition of the mixture and removing the supernatant. Washing was performed at a volume ratio of 1:20. Next, a mixture of PBS and PMSF (1:100) pre-warmed to 95 °C for 20 min was added to the cells. Then, incubation for 10 min at 95 °C and ultrasound treatment with the Vibra Cell device (Sonics, Newtown, CT, USA) were performed. The program was set as follows: amplitude 80%, 10 s working mode, 10 s rest, 18 cycles. Then, centrifugation at 17,000× *g* for 15 min at 4 °C was performed. The supernatant was collected into new tubes, and the protein concentration in the samples was measured using a Qubit fluorimeter (Invitrogen, USA).

### 2.12. Protein Electrophoresis of the Cellular Fraction of the Strain in a Polyacrylamide Gel

Samples prepared according to the method described in Section 2.11, were equilibrated for concentration and volume, 20 mg of protein was placed per well, and samples were dissolved in 4.65× concentrated application buffer (for 1×: glycerol (10%), β-mercaptoethanol (5%), SDS (2.5%), Tris-HCl (0.0625 M, pH 6.8)) and bromphenol blue so that its concentration in the final volume was 0.4 mg/mL. Pierce™ Prestained Protein MW Marker (Thermo Fisher, USA) was used as a marker.

Pre-prepared solutions were used to prepare the gel: solution A (pH 8.8 adjusted with HCl): Trizma base (1.5 M), SDS (0.1%), H_2_O; solution A′ (pH 6.8 adjusted with conc HCl): Trisma base (0.5 M), SDS (0.1%), H_2_O; solution B: acrylamide (29.2%), methylene bisacrylamide (0.8%), H_2_O. The concentrating gel was prepared as follows: solution A′: 1.5 mL, solution B: 1 mL, H_2_O: 3.5 mL, PSA: 80 µL, TEMED: 15 µL. The separating gel was prepared as follows: solution A′—2.5 mL, solution B′—4.15 mL, glycerol—0.5 mL, H_2_O—2.85 mL, PSA—75 µL, TEMED—15 µL. Thus, the final concentration of polyacrylamide in the concentrating gel is 5% and the separating gel is 12%. 

The gels were run at a constant 12 mA current. Gel fixation was performed in the following solution: ethanol (50%), acetic acid (13%), H_2_O. Staining was performed by heating in the following solution: Coomassie Brilliant Blue G-250 (1.3 g/L), ethanol (27%), acetic acid (13%), H_2_O. Washing was performed with 7% acetic acid under heating.

Polyacrylamide gel electrophoresis was repeated before mass spectrometric analysis. Equal amounts of protein (50 μg) from each experimental condition were taken in assay. Protein concentration was determined using Bradford assay. Sample lysates were supplemented with 4× Laemmli Sample buffer and boiled at 95 °C for 5 min and then loaded on 10% SDS-PAGE with 1 mm spacers. PageRuler™ Prestained Protein Ladder (Thermo Fisher, USA) was used as a marker. After electrophoresis, the gel was not fixed and the area of interest (40–55 kDa) was cut out and layered on 10% separating PAGE, 1.5 mm spacer. The gel fragment was fixed with 0.9% agarose. After electrophoresis, the gel was fixed and stained with silver nitrate.

### 2.13. Trypsinolysis and Mass Spectrometric Analysis for the Identification of Proteins from Gel

The protein bands after 1D-PAGE were excised and washed with 100 μL of 15 mM sodium thiosulfate and 50 mM potassium hexacyanoferrate (III) mixture for 10 min at room temperature, and they were washed twice with mQ until the piece of gel becomes transparent. Protein cysteine bonds were reduced with 10 mM DTT in 50 mM NH_4_HCO_3_ for 30 min at 56 °C and alkylated with 55 mM iodoacetamide in the dark at RT for 30 min. The step with adding DTT was repeated. Then, gel pieces were dehydrated with 100 μL of acetonitrile, air-dried and treated by 20 μL of 15 mg/mL solution of trypsin (Trypsin Gold, Mass Spectrometry Grade, Promega) in 50 mM ammonium bicarbonate for 16 h at 37 °C. Peptides were extracted with 50 μL of 50% acetonitrile (ACN)/5% formic acid (FA) solution for 30 min with sonication. This extraction procedure was repeated 3 times. Peptides were dried in vacuum and redissolved in 5% ACN with 0.1% FA solution prior to LC-MS/MS analysis. A total of 5 μL of each sample were injected in trap-elute manner on trap column cartridge (PepMap Neo C18 5 μM 300 μm × 5 mm, Thermo Scientific, Waltham, MA, USA) with 10 μL/min flow rate of loading solution. Peptides were separated on capillary column (Peaky, Reprosil Pur C18 AQ 1.9 μM, 75 μm × 50 cm, «Molecta», Moscow, Russia). Elution was performed with mobile phase gradient from 5% of solution B (80% acetonitrile, 0.1% formic acid) in solution A (0.1% formic acid) at flow rate of 250 nL/min to 65% of solvent B in 60 min.

Detection was performed in data-dependent acquisition mode on high-resolution quadrupole-orbitrap tandem mass-spectrometer Exploris 480 (Thermo Scientific, Waltham, MA, USA). Electrospray Nanospray Flex NG ion source was used for ionization with voltage set at 2200 volts. Precursor scan was performed for ions with *m*/*z* from 200 to 1500 at resolution 60,000 (at 200 *m*/*z*) with internal mass calibration by fluoranthene ions introduced through EASY-IC ion source. Up to 30 precursors with charge from 2 to 6 were subjected to fragment ion scan (resolution 15,000 at 200 *m*/*z*) with normalized collision energy set to 30%.

The obtained data were processed using MaxQuant v 2.4.2.0 software.

### 2.14. Statistical Analysis

Data for comparison were presented as mean ± SD (standard deviation). Statistical significance of the results was assessed using the non-parametric Kruskal–Wallis test. A *p*-value less than 0.05 was considered statistically significant. Statistical analysis was performed using GraphPad Prism 9.0 software (GraphPad, San Diego, CA, USA) and R (v3.5.2)/RStudio [39].

## 3. Results

### 3.1. Survival of Danio rerio Individuals Exposed to Bisphenol A

**Figure 1:**
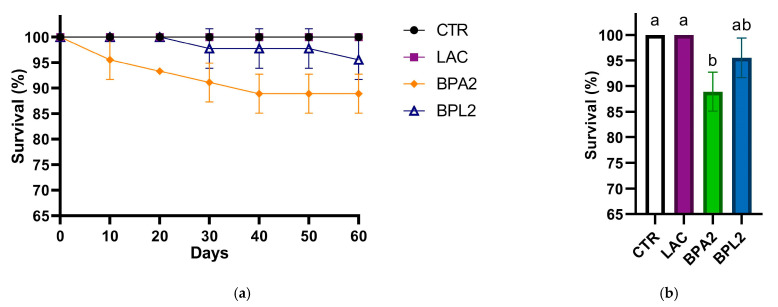
(**a**) Survival dynamics of *Danio rerio* individuals for 60 days and (**b**) on day 60 of the experiment to study the survival of individuals when exposed to bisphenol A in the presence/absence of probiotic. CTR—control group, LAC—group received *L. brevis* 47f with feed and were in a toxicant-free solution, BPA2—group received basic feed and were in a bisphenol A solution at a concentration of 2 mg/L, BPL2—group received *L. brevis* 47f under consideration with feed and were in a bisphenol A solution at a concentration of 2 mg/L. According to Kruskal–Wallis test, *p*-value < 0.05. Letters above the columns represent the static significance between the different experimental groups.

On day 60 of the experiment, the survival rate of individuals exposed in bisphenol A solution and fed probiotic (BPL group) was 95.6 ± 1.8%, and that of individuals not fed probiotic (BPA group) was 88.8 ± 1.7%. No significant changes were found between the other groups (Figure 1).

### 3.2. Measurement of Relative Cytokine Gene Expression Levels in Intestinal Tissues of Danio rerio Individuals Exposed to Bisphenol A 

**Figure 2:**
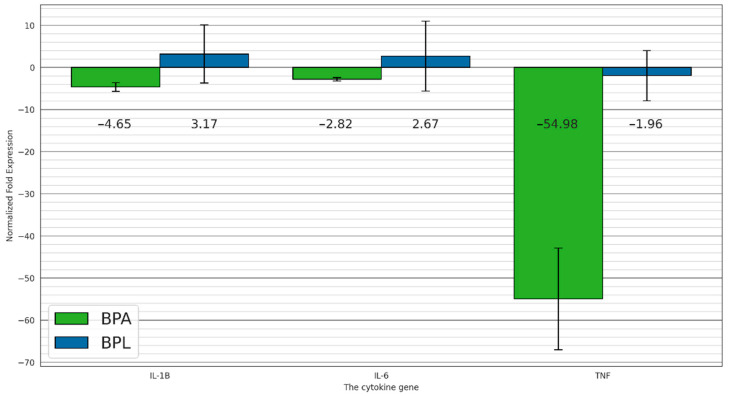
Relative expression level of some cytokines in *Danio rerio* intestinal tissues in bisphenol A solution with a concentration of 2 mg/L depending on the fact that the *Levilactobacillus brevis* 47f strain was received.

The following experimental groups of individuals were compared: BPL—a group in bisphenol A solution and fed a probiotic, BPA—a group also in bisphenol A solution but fed without additives. Bisphenol A exposure in *Danio rerio* intestinal tissues significantly reduced the expression of the pro-inflammatory cytokine genes *il-1b, il-6,* and cytokine gene *tnf-α* by 4.65-, 2.82-, and 54.98-fold, respectively. Addition of *Levilactobacillus brevis* 47f strain to the feed of *Danio rerio* still in bisphenol A solution increases the expression of cytokine genes *il-6* and *il-1b* by 2.67-, and 3.17-fold, respectively, and decreases the expression of *tnf-α* by 1.96-fold. *Levilactobacillus brevis* 47f strain reduces the effect of bisphenol A on *tnf-α* cytokine gene expression by 28-fold (Figure 2). 

### 3.3. Analysis of Differential Gene Expression of Levilactobacillus brevis 47f Strain under Exposure to Different Doses of Bisphenol A 

**Figure 3:**
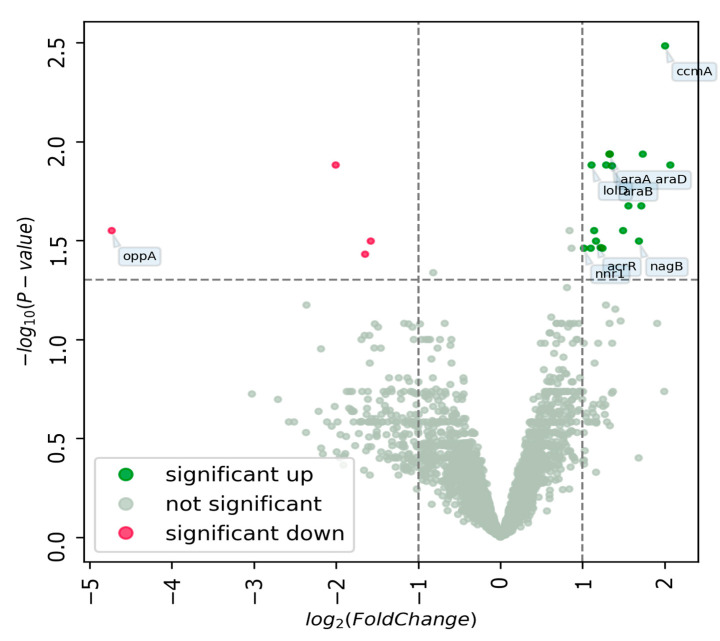
Volcano plot showing *L. brevis* 47f genes whose expression change is statistically significant at a bisphenol A concentration of 50 mg/L (*p*-value < 0.05, |Fold Change| ≤ 2).

The minimum inhibitory concentration (MIC) of bisphenol A when exposed to the *L. brevis* 47f strain under study was 100 mg/L. To investigate the dose-dependent effect of the toxicant on the *L. brevis* 47f strain in the experiment, the following concentrations with no effect on the strain growth were chosen: 2, 50 mg/L (½ MIC). As a result of the analysis, genes whose relative expression was statistically significant were identified (Figure 3, Table 1). No statistically significant change in gene expression was found at a bisphenol A concentration of 2 mg/L. 

Noteworthy is the increase in the expression of *araA*, *araB*, and *araD* genes located in the same operon and involved in arabinose metabolism. There is also a change in the expression of genes of various transport proteins, genes involved in the synthesis of fatty acids, and genes of transcriptional regulators, in addition to the *oppA* gene encoding a protein responsible for the attachment of bacterial cells to the surfaces of mucous membranes [40]. According to literature data, this gene is also capable of influencing stress resistance [41].

The *lolD* gene product is part of the LolCDE transport complex involved in lipoprotein relocalization [42]. The *acrR* gene product is involved in the regulation of fatty acid synthesis and membrane fluidity, which contributes to stress adaptation [43]. The *nagB* gene product catalyzes the reversible isomerization-deamination of glucosamine-6-phosphate (GlcN6P) to form fructose-6-phosphate (Fru6P) and ammonium ion, and it is involved in processes of resistance to acid stress [44]. Among the regulators of transcription, regulators of the GntR family have been identified that control such processes as cell motility, glucose metabolism, bacterial resistance, pathogenesis and virulence [45]. The *nnr1* gene product participates in the reduction of NAD(P)H hydrates, which are damage products [46]. 

### 3.4. Protein Electrophoresis in Polyacrylamide Gel of Cell Fraction of Levilactobacillus brevis 47f upon Exposure to Different Doses of Bisphenol A 

**Figure 4:**
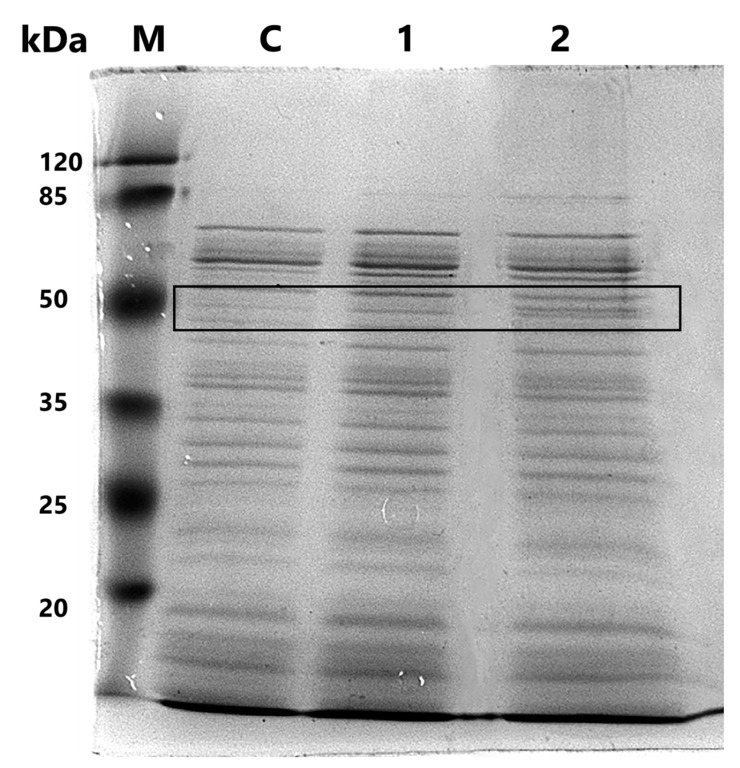
Electrophoregram of *Levilactobacillus brevis* 47f cell fraction proteins when treated with different concentrations of bisphenol A (C, 1, 2–0, 2, 50 mg/L, respectively). M marker (120, 85, 50, 35, 25, 20 kDa). The arrow indicates the protein fraction to be analyzed. The original gel can be found in Supplementary Figure S1.

Polyacrylamide gel electrophoresis of the cellular protein fraction of the *L. brevis* 47f strain revealed a dose-dependent change in line intensity in the region of 40–55 kDa (Figure 4), indicating possible proteins involved in the demonstration of adaptogenic properties by the *L. brevis* 47f strain or in its stress response. This band was analyzed by mass spectrometry to identify the proteins.

### 3.5. Identification of Proteins from the Gel by Mass Spectrometry Analysis

Mass spectrometric analysis to identify proteins in the band of interest in the electrophoregram revealed the following proteins is presented in Table 2.

L-arabinose isomerase was detected in the spectrum of identified proteins, the gene of which increased its expression under conditions of high concentrations of bisphenol A in the full transcriptome analysis as well. In addition, enzyme proteins involved in the pentose phosphate pathway and glycolysis were also detected. A subunit of the Clp chaperone has also been found, which, according to literature data, has a disaggregating function [47]. The number of ATP synthase subunits produced increases with increasing concentration of the toxicant present during cell growth. The increase in the amount of these produced proteins is possibly related to the *L. brevis* 47f strain’s own defense against stressful conditions.

## 4. Discussion

Due to increased environmental pollution with xenobiotics, there is currently an active search for substances capable of leveling the deleterious effects of non-native chemicals on living organisms, in particular aquaculture. It has been shown that prolonged exposure to low doses of xenobiotics present in reservoirs leads to body stress and various physiological and morphological changes [2,4,48]. Probiotics are often used in aquaculture because of their ability to maintain the immune status of individuals and increase their survival rate [19]. An emerging trend in the field of probiotics and functional feed additives is the development of pharmabiotics, the distinctive feature of which is proven safety and a known mechanism of action [10,13]. In this work, the strain *Leivilactobacillus brevis* 47f was tested as an adaptogen for aquaculture, and the effect of bisphenol A on the main parameters of vital activity was evaluated.

It was shown that additives of the *Levilactobacillus brevis* 47f strain led to an increase in the survival rate of *Danio rerio* individuals in groups exposed to bisphenol A (Figure 1). Survival rates for groups treated with bisphenol A solution with the addition of *L. brevis* 47f and without *L. brevis* 47f were 95.6 ± 1.8% and 88.8 ± 1.7%, respectively. Such differences in parameters are significant in aquaculture. An analysis of the relative expression levels of pro-inflammatory and anti-inflammatory cytokines in the intestinal tissues of *Danio rerio* exposed to low doses of xenobiotic showed that the effect of bisphenol A leads to a multidirectional change in their expression levels (Figure 2). Bisphenol A has the most significant effect on the expression of *il-1b*, *il-6* and *tnf-α*. In addition, the *Levilactobacillus brevis* 47f strain reduces the effect of bisphenol A on the expression of the *tnf-α* cytokine gene by 28 times. TNF-a can exhibit both pro-inflammatory and anti-inflammatory properties, and TNF-R2 is involved in anti-inflammatory properties in addition to TNF-a itself: it reduces the synthesis of IL-12, while TNF-R2 increases the secretion of TGF-β [49]. Therefore, in this study, a decrease in the expression level of the gene of cytokine *tnf-a* probably indicates a decrease in its expression as an anti-inflammatory cytokine. The result suggests that the *L. brevis* 47f strain indeed has adaptogenic properties when exposed to low doses of xenobiotics in aquaculture.

In addition to studying the changes occurring in *Danio rerio* under the combined influence of toxicants and probiotic strain, the *L. brevis* 47f strain itself, also grown in the presence of xenobiotics, was studied to identify how critical the bisphenol A effect on the *L. brevis* 47f strain is. Nowadays, omics technologies are actively used for complex study of molecular mechanisms of action of certain bacterial strains. Thus, the study of the transcriptomic and proteomic profile of the *L. brevis* 47f strain grown in the presence of the toxicant bisphenol A allowed us to elucidate whether the toxicant has an effect on the *L. brevis* 47f strain. A comparative analysis of transcriptomic data revealed a number of genes that significantly change their expression when exposed to high doses of bisphenol A (Figure 3). The products of these genes can participate in various metabolic pathways, including arabinose metabolism, fatty acid synthesis, transporters, transcriptional regulators (Table 1). The *oppA* gene, which can influence stress tolerance [41], and whose protein is adhesive to mucous membranes [40], also changes its expression significantly. The products of the genes *araA*, *araD*, *oppA*, *lolD*, *nagB*, *ccmA*, and *nnr1*, whose relative expression changes are significant, are possibly involved in the stress response properties of the *L. brevis* 47f strain. 

Polyacrylamide gel electrophoresis of the cellular fraction of *L. brevis* 47f strain proteins showed an increase in the intensity of the protein bands in the region of the 50 kDa at a bisphenol A concentration of 50 mg/L (Figure 4), which may indicate the activation of anti-stress mechanisms of the strain, allowing it to adapt to the action of the toxicant. Identification of the protein spectrum by LC-MS/MS analysis (Table 2) revealed a dose-dependent increase in the amount of such proteins as L-arabinose isomerase, ATP-dependent subunit of Clp chaperone, ATP synthase subunit, pentose phosphate pathway and glycolysis enzyme proteins. L-arabinose isomerase, whose gene was also detected by transcriptome analysis, is responsible for the conversion of arabinose to ribulose, which, subject to phosphorylation modification, is one of the products of the pentose phosphate pathway [50]. Both glycolysis and the pentose phosphate pathway lead to the production of free ATP. However, large amounts of extracellular ATP can serve as a signal to attract pro-inflammatory cytokines and the cell goes into apoptosis [51]. But at the same time, the chaperone Clp, its subunit, is actively secreted. The normal work of chaperones requires ATP, so, most likely, the generated ATP goes to the work of chaperones, which are activated under stress [52]. One of the functions of Clp is disaggregating, so they can contribute to the condition of both the bacterial cell and the host organism [47,53]. They also dissolve loose protein aggregates, bind excessively damaged proteins into larger aggregates and direct severely damaged proteins to degrade [54].

The results obtained demonstrate a significant increase in the survival rate of *Danio rerio* in the presence of bisphenol A when exposed to the *L. brevis* 47f strain. Significant changes in the transcriptomic and proteomic profile of the *L. brevis* 47f strain were also recorded at high concentrations of bisphenol A, which are achievable only in laboratory conditions. This allows us to suggest that when conducting a study to find the mechanism of action of the *L. brevis* 47f strain, we can ignore the effect of the toxicant on the strain itself, its proteomic and transcriptomic profile. In this way, we can consider only those proteins, metabolites of the *L. brevis* 47f strain, that are produced in the absence of toxicant.

Thus, the *Levilactobacillus brevis* 47f strain can be used in aquaculture as an adaptogen to low doses of xenobiotics. Bisphenol A, in its turn, has no significant effect on the vital activity and protein spectrum of the *L. brevis* 47f strain. In the future, additional studies are planned to identify the possible mechanism of action of the *L. brevis* 47f strain as an adaptogen using omics technologies.

## 5. Conclusions

The strain *Levilactobacillus brevis* 47f is capable of exhibiting protector properties in aquaculture, serving as an adaptogen when exposed to low doses of xenobiotics in aquaculture. There was a significant increase in the survival rate of individuals in the presence of bisphenol A and receiving the *L. brevis* 47f strain compared to individuals not fed the supplement. A significant change in the cytokine profile in the intestinal tissues of *Danio rerio* was also observed. Transcriptome analysis of the *L. brevis* 47f strain in the presence of high concentrations of bisphenol A allowed us to identify genes that significantly change their expression under stress. Among the identified genes, a number of them are involved in adaptation to various types of stress. Proteins with chaperone activity, glycolysis proteins, and pentose phosphate pathway proteins were identified by proteomic analysis. The increase in the number of proteins at high concentrations of the bisphenol A is more likely to indicate the very anti-stress defense of the *L. brevis* 47f strain itself rather than its ability to exhibit adaptogenic properties in *Danio rerio* organism. Further application of metabolomic analysis and proteomic analysis of the whole spectrum of proteins produced will allow to clarify the mechanism of action of the *Levilactobacillus brevis* 47f.

## Figures and Tables

**Table 1:** Investigated *L. brevis* 47f genes whose change in expression is statistically significant upon exposure to bisphenol A at a concentration of 50 mg/L.

Geneid	Product	Gene	Fold Change	FDR
AAX72_RS00490	L-arabinose isomerase	*araA*	2.508	0.012
AAX72_RS00495	L-ribulose-5-phosphate 4-epimerase	*araD*	2.517	0.012
AAX72_RS00500	FGGY-family carbohydrate kinase	*araB*	2.562	0.013
AAX72_RS02630	oligopeptide ABC transporter substrate-binding protein	*-*	2.205	0.028
AAX72_RS05195	peptide ABC transporter substrate-binding protein	*oppA*	−26.556	0.028
AAX72_RS04190	ABC transporter ATP-binding protein	*lolD*	2.157	0.013
AAX72_RS07190	TetR/AcrR family transcriptional regulator	*acrR*	2.240	0.032
AAX72_RS07195	ABC transporter ATP-binding protein/permease	*-*	2.945	0.021
AAX72_RS07520	glucosamine-6-phosphate deaminase	*nagB*	3.218	0.032
AAX72_RS08605	ABC transporter ATP-binding protein	*ccmA*	4.012	0.003
AAX72_RS08610	GntR family transcriptional regulator	*-*	4.193	0.013
AAX72_RS09615	NAD(P)H-hydrate epimerase	*nnr1*	2.023	0.035

**Table 2:** Spectrum of identified proteins in the target band of the electrophoregram as a function of bisphenol A concentration (0 mg/L (C), 2 mg/L, 50 mg/L). Numerical values represent LFQ Intensity reduced by a factor of 10^6^.

Protein	C	2 mg/L	50 mg/L	Functions and Biochemical Pathways
L-arabinose isomerase	0.0	6.8	21.8	Conversion of L-arabinose to L-ribulose
catabolite control protein A	10.0	31.5	27.4	Transcriptional regulator of carbon catabolism
gluconokinase	0.0	21.4	32.5	The pentose phosphate pathway
phosphopentomutase	0.0	17.4	63.9	
NADP-dependent phosphogluconate dehydrogenase	20.4	23.5	79.3	
glucose-6-phosphate dehydrogenase	0.0	23.8	16.5	
type I glyceraldehyde-3-phosphate dehydrogenase	36.8	144.5	235.7	Glycolysis
phosphoglycerate kinase	1745.0	1232.9	1344.8	
glucose-6-phosphate isomerase	24.0	52.8	45.0	
phosphopyruvate hydratase	217.8	279.6	484.5	
elongation factor Tu	819.8	890.0	1640.1	Binding of aminoacyl-tRNA to the A-site of the ribosome
peptide chain release factor 1	20.4	6.3	27.9	Translation
trigger factor	21.0	25.1	46.6	The first chaperone that interacts with the newly synthesized polypeptide chain as it leaves the ribosome into the cytoplasm
ATP-dependent Clp protease ATP-binding subunit	21.3	22.0	34.6	Chaperone subunit
30S ribosomal protein S1	28.0	717.2	591.0	Translation, mRNA binding to the 30S subunit of the ribosome, control of RNA stability
F0F1 ATP synthase beta subunit	0.0	26.7	33.3	ATP synthesis
F0F1 ATP synthase alpha subunit	0.0	0.0	37.1	
serine hydroxymethyltransferase	6.9	10.7	46.3	Serine metabolism
serine-tRNA ligase	0.0	0.0	21.2	

## Data Availability

The transcriptomic data have been uploaded to NCBI (BioProject PRJNA1121097).

## Supplementary Materials

The following supporting information can be downloaded at: https://www.mdpi.com/article/10.3390/biology13110925/s1, Figure S1: Original gel of *Levilactobacillus brevis* 47f cell fraction proteins when treated with different concentrations of bisphenol A (C, 1, 2–0, 2, 50 mg/L, respectively). M marker (120, 85, 50, 35, 25, 20 kDa).

## Appendix A

**Table A1:** Primers used in real-time PCR.

Target Gene	R/F *	Nucleotide Sequence (5′→3′)	NCBI RefSeq
*il-1b*	F	CATTTGCAGGCCGTCAC	NM_212844.2
	R	GGACATGCTGAAGCGCACT	
*tnf-α*	F	GCTGGATCTTCAAAGTCGGGTGT	NM_212859.2
	R	TGTGAGTCTCAGCACACTTCCAT	
*ifnphi1*	F	GAATGGCTTGGCCGATACAGGAT	NM_207640.1
	R	TCCTCCACCTTTGACTTGTCCAT	
*il-6*	F	TCAACTTCTCCAGCGTGAT	NM_001261449.1
	R	TCTTTCCCTCTTTTCCTCCT	
*il-8*	F	GTCGCTGCATTGAAACAGA	XM_001342570.7
	R	CTTAACCCATGGAGCAGAG	
*il-10*	F	CCCTATGGATGTCACGTCAT	NM_001020785.2
	R	CATATCCCGCTTGAGTTCCT	
*actb1*	F	ATGGATGAGGAAATCGCTGCC	NM_131031.2
	R	CTCCCTGATGTCTGGGTCGT	

* F—forward sequence, R—reverse sequence.
