# Supplementary material for: Levilactobacillus brevis 47f: Bioadaptation to Low Doses of Xenobiotics in Aquaculture
Source: Biology (Basel). 2024 Nov 14;13(11):925. doi: 10.3390/biology13110925 (PMC11592329; doi:10.3390/biology13110925)
Supplement: Supplementary file 1 [file biology-13-00925-s001.zip › biology-3293511-supplementary.pdf]

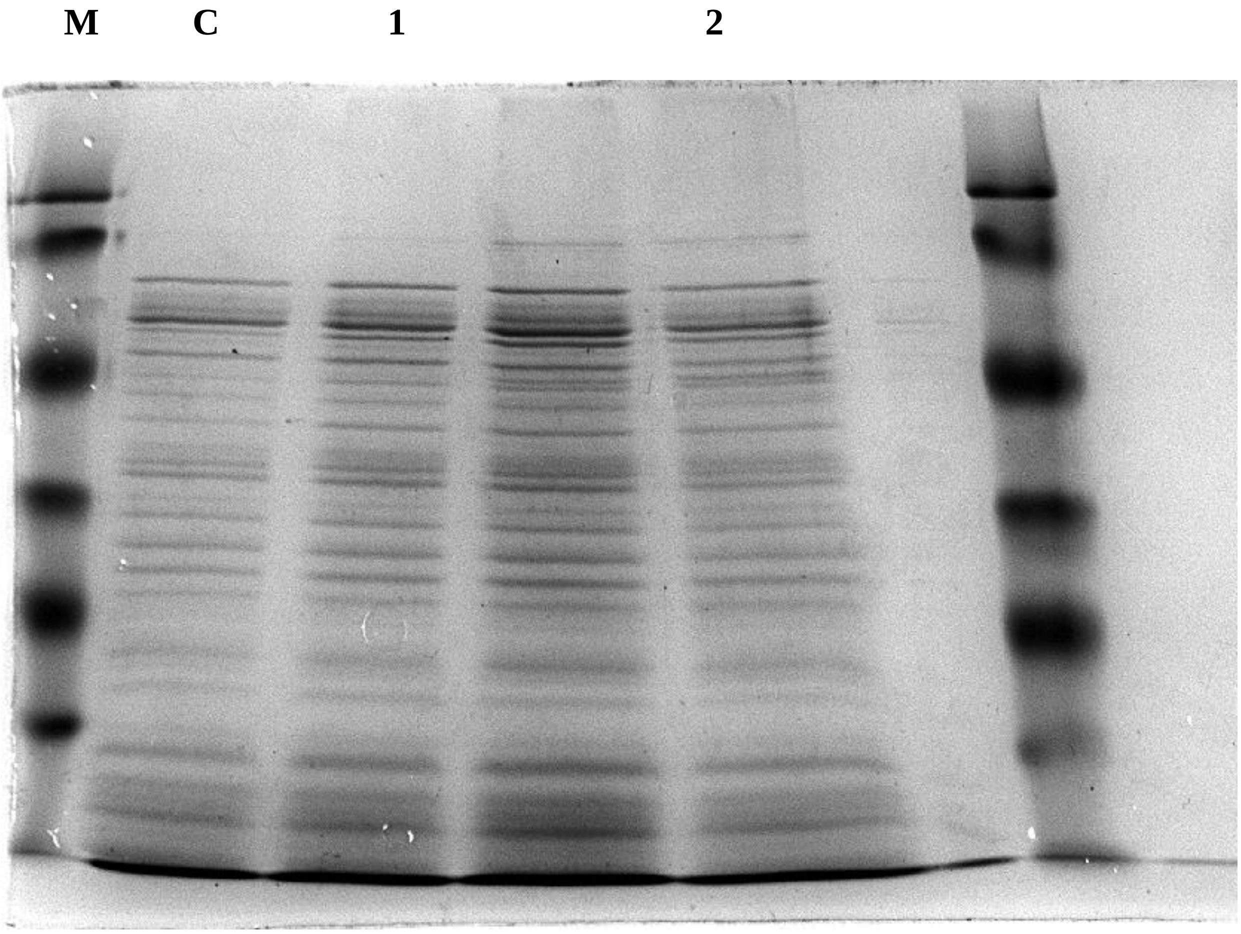

Figure S1. Original gel of *Levilactobacillus brevis* 47f cell fraction proteins when treated with different concentrations of bisphenol A (C, 1,2 – 0, 2, 50 mg/L, respectively). M marker (120, 85, 50, 35, 25, 20 kDa).
